# Association between breakfast consumption and educational outcomes in 9–11-year-old children

**DOI:** 10.1017/S1368980015002669

**Published:** 2015-09-28

**Authors:** Hannah J Littlecott, Graham F Moore, Laurence Moore, Ronan A Lyons, Simon Murphy

**Affiliations:** 1 Centre for the Development and Evaluation of Complex Interventions for Public Health Improvement (DECIPHer), School of Social Sciences, Cardiff University, 1–3 Museum Place, Cardiff CF10 3BD, UK; 2 MRC/CSO Social and Public Health Sciences Unit, University of Glasgow, Glasgow, UK; 3 Centre for the Development and Evaluation of Complex Interventions for Public Health Improvement (DECIPHer), Centre for Health Information, Research and Evaluation, Swansea University, Swansea, UK

**Keywords:** Breakfast consumption, Educational outcomes, Socio-economic inequalities, Free school breakfast

## Abstract

**Objective:**

Breakfast consumption has been consistently associated with health outcomes and cognitive functioning in schoolchildren. Evidence of direct links with educational outcomes remains equivocal. We aimed to examine the link between breakfast consumption in 9–11-year-old children and educational outcomes obtained 6–18 months later.

**Design:**

Data on individual-level free school meal entitlement and educational outcomes (Statutory Assessment Tests (SATs) at Key Stage 2) were obtained via the SAIL databank and linked to earlier data collected on breakfast consumption. Multilevel modelling assessed associations between breakfast consumption and SATs.

**Setting:**

Trial of the Primary School Free Breakfast Initiative in Wales.

**Subjects:**

Year 5 and 6 students, *n* 3093 (baseline) and *n* 3055 (follow-up).

**Results:**

Significant associations were found between all dietary behaviours and better performance in SATs, adjusted for gender and individual- and school-level free school meal entitlement (OR=1·95; CI 1·58, 2·40 for breakfast, OR=1·08; CI 1·04, 1·13 for healthy breakfast items). No association was observed between number of unhealthy breakfast items consumed and educational performance. Association of breakfast consumption with educational performance was stronger where the measure of breakfast consumption was more proximal to SATs tests (OR=2·02 measured 6 months prior to SATs, OR=1·61 measured 18 months prior).

**Conclusions:**

Significant positive associations between self-reported breakfast consumption and educational outcomes were observed. Future research should aim to explore the mechanisms by which breakfast consumption and educational outcomes are linked, and understand how to promote breakfast consumption among schoolchildren. Communicating findings of educational benefits to schools may help to enhance buy-in to efforts to improve health behaviours of pupils.

Interventions to improve child health are increasingly delivered via the school setting, in part because this provides opportunities to reach large numbers of children simultaneously. Such interventions range from health education programmes^(^ ^1^ ^)^ to holistic settings-based approaches^(^ ^2^ ^)^. Reviews indicate that interventions based on education alone are typically ineffective^(^ ^3^ ^)^, while complex interventions including components at multiple levels often have greater overall effects^(^ ^4^ ^)^. Hence, effective interventions often require significant changes to the structures and practices of schools. However, directing time and resources towards improving child health is commonly seen as diverting time away from schools’ core business of educating pupils, in part due to pressures from sources including regulatory bodies that emphasise educational attainment^(^ ^5^ ^)^.

However, this resistance to delivery of health improvement interventions overlooks the potential synergy between health and education, and the growing evidence that improving health may also improve educational outcomes. In the UK, there is a growing policy emphasis on the need to understand links between health and education agendas in schools, in order to align such programmes with the ‘core business’ of the school and achieve buy-in^(^ ^5^ ^)^. For example, Public Health England has produced a briefing for school staff highlighting the reciprocal relationship between pupil health and well-being and attainment, emphasising the importance of a whole-school approach^(^ ^6^ ^)^. Moreover, recent education reviews in Wales and Scotland have highlighted physical and mental health and well-being as a priority^(^ ^7^ ^)^.

The evidence base linking health behaviours to educational outcomes is at present underdeveloped; there is little prospective evidence, while few public health researchers evaluate the impacts of their interventions on education^(^ ^8^ ^)^. However, strong cross-sectional associations have been found between educational outcomes and health behaviours^(^ ^9^ ^)^, such as physical activity^(^ ^10^ ^)^ and substance use^(^ ^11^ ^)^. Links between educational outcomes and dietary factors remain equivocal, in part due to the difficulty defining, identifying and measuring the key dietary factors^(^ ^12^ ^)^.

One dietary behaviour for which effects on education are often presumed, and which is commonly targeted by school-based interventions, is breakfast consumption. In one UK survey of fifty-nine schools, Hoyland *et al*.^(^ ^13^ ^)^ found that 14 % of 7–15-year-old children reported skipping breakfast, with one-third reporting that they did not eat anything until lunchtime. Breakfast consumption is also socially patterned, thus putting less affluent children at a higher risk of experiencing negative health outcomes associated with skipping breakfast^(^ ^14^ ^,^ ^15^ ^)^. In the UK, breakfast clubs were offered in almost half of schools in England by 2012, with delivery concentrated in areas of deprivation^(^ ^13^ ^)^. In Wales, the Welsh Government’s Primary School Free Breakfast Initiative was implemented from 2005^(^ ^16^ ^,^ ^17^ ^)^.

However, while breakfast has been consistently associated with health outcomes^(^ ^12^ ^,^ ^18^ ^)^, evidence regarding links to educational outcomes remains equivocal. While a body of research examines impacts of breakfast consumption on acute cognitive performance in terms of episodic memory, visual searching and concentration^(^ ^19^ ^–^ ^22^ ^)^, fewer studies have examined associations with direct measures of educational performance. In their review of breakfast habits and academic performance, Rampersaud *et al*.^(^ ^12^ ^)^ found significant improvements in achievement test scores for Jamaican children who consumed breakfast during two randomised controlled trials. A further randomised controlled trial found improved attendance rates for Peruvian children in the school breakfast arm^(^ ^20^ ^)^. They concluded that future trials should investigate the longitudinal effects of breakfast consumption on academic outcomes and control for socio-economic status (SES)^(^ ^12^ ^)^. A recent review concluded that there remained insufficient evidence for the relationship between breakfast consumption and longer-term attainment^(^ ^23^ ^)^, while Adolphus *et al*.^(^ ^24^ ^)^ reported that few such studies adopt longitudinal designs, adjusted adequately for confounding variables such as SES, or included large samples and validated measures of academic performance. In fact, no previous studies have examined the role of breakfast consumption in mediating SES inequalities in educational outcomes.

The current study uses data collected as part of the cluster-randomised controlled trial of the Welsh Government’s Primary School Free Breakfast Initiative in a large representative UK population, while also examining whether any effect observed differs by SES. A cluster-randomised trial of this scheme demonstrated effects on the quality of children’s breakfast foods and reduction in socio-economic inequality in breakfast skipping, despite a lack of main effect on breakfast skipping^(^ ^25^ ^)^. It was not possible to link the intervention to educational outcomes, as control schools took up the scheme between completion of the trial and collection of educational performance data. However, within the present paper, we examine longitudinal associations between breakfast consumption (in terms of both whether children eat breakfast and the quality of foods children eat for breakfast) and subsequent exam results. A secondary analysis examines whether better educational outcomes were achieved in schools receiving the free school breakfast intervention during the trial period. The following research questions were addressed:

1. Is there a link between breakfast consumption and other dietary behaviours and educational outcomes in 9–11-year-old children?
2. Did any existing relationship differ in strength according to the proximity of the dietary recall measures to the collection of educational outcomes data?
3. Are socio-economic differences in educational performance impacted by SES differences in breakfast consumption?

## Methods

### Sampling and participants

Full details of methods and sampling procedures are described elsewhere^(^ ^16^ ^)^. Primary schools in Wales were approached to take part in a cluster-randomised controlled trial of the Welsh Government’s Primary School Free Breakfast Initiative. A total of 4350 students in Years 5 and 6 (i.e. aged 9–11 years) at baseline and 4472 at 12-month follow-up completed classroom-based attitude and dietary recall questionnaires within the 111 schools that took part. Due to increased class sizes in some schools, the eligible pool of children increased slightly, although response rates were identical at baseline and follow-up (88·3 %). Of the 4350 and 4472 children who completed baseline and follow-up measures, individual-level free school meal (FSM) entitlement and educational outcomes data were obtained for 3093 (71·1 %) and 3055 (68·3 %). The trial used a repeated cross-sectional design, sampling Year 5 and 6 pupils at baseline and follow-up. However, a nested cohort of 1216 children (who were in Year 5 at baseline but Year 6 at follow-up) provided data at both baseline (16–18 months prior to collection of educational outcomes data in May) and follow-up (4–6 months prior to collection of educational outcomes data in May). A secondary analysis therefore examines links between reported breakfast consumption at these two time points and educational performance tests among this sub-sample.

### Measures

#### Deprivation

This was assessed using data on FSM entitlement, including a school-level measure (percentage of children entitled to FSM within the whole school) and children’s own entitlement to FSM (yes or no).

#### Dietary recall questionnaire

The questionnaire was a modified version of the Day in the Life Questionnaire^(^ ^26^ ^)^. This measure has been validated against 24 h recall interviews with a sub-sample of children from the present study and offers an acceptable level of validity and reliability^(^ ^27^ ^)^. The measure covered a period slightly in excess of 24 h; children were asked to list all foods and drinks consumed at chronologically ordered time points throughout the previous day and for breakfast on the day of reporting. Outcome variables are the proportion of children consuming less than two breakfasts over 2 d, the number of healthy items (i.e. cereals, bread, fruits and milk products) consumed for breakfast, the number of unhealthy items (i.e. crisps and sweet snacks) consumed for breakfast, the number of fruit and vegetable portions consumed during the rest of the day and the number of unhealthy items (i.e. crisps and sweet snacks) consumed during the rest of the day.

#### Statutory Assessment Tests

Following the trial, children’s reports of their breakfast consumption behaviours were linked to their scores on Key Stage 2 Statutory Assessment Tests (SATs). SATs are mandatory tests taken by all Year 6 (aged 10–11 years) students in Wales and England in mid-May. In 2005, when this trial was conducted, students were assessed in English, Maths and Science and graded with levels 1–6.

### Procedure

For the original trial, parents were informed of the research by means of a letter and information sheet sent home with children and were asked to contact the school if they did not wish their child to participate in the study. Parents of fifteen children requested that their child be excluded. At each data collection, children were also informed that they were under no obligation to participate. Class-level measures were completed in the morning (between 09.00 and 11.00 hours) as a supervised classroom exercise with a maximum class size of forty children. For the dietary recall measure and the cognitive measures, the researcher read out the instructions and asked children to complete the task independently from one another. If children had finished or needed help with spelling or further clarification, they were asked to put their hands up. Three members of the research team were present to assist children. The main trial design and results are presented in detail elsewhere^(^ ^16^ ^,^ ^28^ ^)^. The SAIL (Secure Anonymised Information Linkage) databank is a data warehouse that was established at the Health Information Research Unit at the College of Medicine at Swansea University. It brings together a wide range of person-based data, using a split-file approach to anonymisation to overcome issues of confidentiality and disclosure^(^ ^29^ ^)^, and operates within a robust series of guidelines in line with the Caldicott principles and the National Information Governance Board for Health and Social Care^(^ ^30^ ^)^. Participating children were each assigned an Anonymised Linking Field (ALF_E)^(^ ^29^ ^)^ and thereby linked to the National Pupil Database for Wales, a version of which is held on the SAIL databank. This allowed the dietary data to be linked to the SATs and individual FSM entitlement data for each participant.

### Statistical analysis

Descriptive statistics were conducted to describe the characteristics of participants. Mixed-effects binary logistic regression models, with pupils nested within the school, were used to assess the association between breakfast consumption and SATs results. A *P* value of 0·05 was considered a significant association. Whole-sample analyses were conducted using separate models for baseline and follow-up samples on dietary behaviour. Primary analysis focused on the baseline data, which were collected before any intervention related to breakfast was implemented. The first model included individual-level demographics (gender and FSM entitlement), while the second model combined individual-level demographics and school-level FSM entitlement. Being male and being ineligible for FSM were used as the reference groups for the independent variables within the multilevel models (i.e. male=0, female=1), whereas for school-level FSM entitlement, a higher score=a higher percentage of pupils entitled to FSM (i.e. higher deprivation). Subsequent models added terms for each breakfast consumption measure: ‘number of healthy breakfast items consumed’, ‘number of unhealthy breakfast items consumed’, ‘number of sweets and crisps consumed’ or ‘number of fruit and vegetable portions consumed’. These analyses were run twice for the nested cohort (*n* 1216), first examining links between breakfast consumption at baseline and educational performance, and second examining associations of reported behaviour at follow-up. A secondary analysis compared educational outcomes for children in intervention and control schools. Follow-up data collections for the school breakfast trial were completed by April 2006 in Phase 1 of the trial (Communities First schools) and by February 2007 in Phase 2 (after which wait-list controls were free to set up the intervention). Hence, to minimise the risk of contamination, the present analyses include only children taking their SATs in May 2006 for Communities First schools and in 2007 in non-Communities First schools (i.e. those children in Year 6 when the trial ended). All analyses were conducted using the statistical software package Stata version 13.

## Results

The samples are described in Table 1. The nested cohort was comparable to the full sample in terms of FSM entitlement, gender, breakfast consumption behaviours and educational outcomes.

**Table 1:** Descriptive statistics of the samples of Year 5 and 6 students aged 9–11 years participating in the cluster-randomised controlled trial of the Welsh Government’s Primary School Free Breakfast Initiative

	Whole sample (follow-up only)				Cohort (*n* 1216)			
	Baseline (*n* 3093)		Follow-up (*n* 3055)		Baseline		Follow-up	
	*n* or median	% or IQR	*n* or median	% or IQR	*n* or median	% or IQR	*n* or median	% or IQR
FSM entitlement*	674	21·8	706	23·1	263§		21·6||	
Girls	1571	50·8	1511	49·5	643		52·9	
Ate two breakfasts in 2 d	2459	79·5	2506	82·0	984	80·9	988	81·3
No. of healthy items for breakfast†	3	2–4	4	2–5	3	2–4	2	3–5
At least one ‘unhealthy’ breakfast item	666	21·5	552	18·1	264	21·7	224	18·4
At least one fruit or vegetable (rest of day)	1404	45·4	1512	49·5	521	42·8	570	47·4
At least one sweet or crisp item (rest of day)	2102	68·0	1957	64·1	804	66·1	793	70·0
English (% scoring 4 or 5)‡	2433	78·6	2401	78·6	987		80·8	
Maths (% scoring 4 or 5)‡	2489	80·4	2444	80·0	993		81·7	
Science (% scoring 4 or 5)‡	2683	86·7	2628	86·0	1081		88·5	

* FSM entitlement refers to those students from low-income families who are, therefore, eligible to receive free school meals at lunchtime.

† Data are presented as median and interquartile range (IQR).

‡ In 2005, when this trial was conducted, students were assessed in English, Maths and Science and graded with levels 1–6.

§ Data (all such values) are numbers.

|| Data (all such values) are percentages.

### Dietary behaviours and educational outcomes

Table 2 shows the results of the model using the baseline and follow-up samples. Being female, not being entitled to FSM and a lower school-level FSM entitlement were all independently associated with higher SATs scores. The subsequent models, which included dietary behaviours, showed that breakfast consumption, number of healthy breakfast items consumed for breakfast, number of sweets and crisps consumed throughout the rest of the day and number of fruit and vegetable portions consumed throughout the rest of the day, adjusted for gender and individual- and school-level FSM entitlement, were all significantly and positively associated with educational performance. No association was observed between the number of unhealthy breakfast items consumed and educational performance. Associations of school- and individual-level measures of SES with education remained unchanged after inclusion of dietary variables, providing no evidence that their association with educational performance was mediated by SES differences for any outcome. The intra-cluster correlation coefficient declined from 0·8 in the model containing only individual-level demographic variables to 0·7 in models containing school-level FSM and dietary variables, indicating that only a small proportion of school-level variance is explained by these additional variables.

**Table 2:** Odds ratios and 95 % confidence intervals from multilevel binary logistic regression analysis using individual- and school-level demographics and baseline (*n* 3093) and follow-up data (*N* 3055) to investigate the associations between dietary behaviours and improved educational outcomes among Year 5 and 6 students aged 9–11 years participating in the cluster-randomised controlled trial of the Welsh Government’s Primary School Free Breakfast Initiative

	Baseline (*n* 3093)		Follow-up (*n* 3055)		
Variable	OR	95 % CI	OR	95 % CI	ICC
Individual-level demographics					
Gender*	**1·55**	**1·30, 1·84**	**1·49**	**1·25, 1·77**	0·08
FSM entitlement†	**0·36**	**0·30, 0·44**	**0·37**	**0·30, 0·44**	
Individual-level demographics & school-level FSM					
Gender	**1·55**	**1·30, 1·84**	**1·50**	**1·26, 1·78**	0·07
FSM entitlement	**0·38**	**0·31, 0·46**	**0·38**	**0·31, 0·46**	
School-level FSM‡	**0·99**	**0·98, 1·00**	**0·99**	**0·98, 1·00**	
Individual-level demographics & school-level FSM & breakfast					
Gender	**1·51**	**1·27, 1·80**	**1·44**	**1·21, 1·71**	0·07
FSM entitlement	**0·39**	**0·32, 0·47**	**0·38**	**0·31, 0·47**	
School-level FSM	**0·99**	**0·98, 1·00**	**0·99**	**0·98, 1·00**	
Breakfast	**1·48**	**1·21, 1·82**	**1·95**	**1·58, 2·40**	
Individual-level demographics & school-level FSM & healthy breakfast items					
Gender	**1·54**	**1·30, 1·83**	**1·48**	**1·24, 1·76**	0·07
FSM entitlement	**0·38**	**0·31, 0·46**	**0·38**	**0·31, 0·46**	
School-level FSM	**0·99**	**0·98, 1·00**	**0·99**	**0·98, 1·00**	
Healthy breakfast items	**1·09**	**1·04, 1·14**	**1·08**	**1·04, 1·13**	
Individual-level demographics & school-level FSM & unhealthy breakfast items					
Gender	**1·55**	**1·30, 1·84**	**1·50**	**1·26, 1·78**	0·07
FSM entitlement	**0·38**	**0·31, 0·46**	**0·38**	**0·32, 0·47**	
School-level FSM	**0·99**	**0·98, 1·00**	**0·99**	**0·98, 1·00**	
Unhealthy breakfast items	1·00	0·88, 1·13	0·95	0·84, 1·07	
Individual-level demographics & school-level FSM & sweets/crisps					
Gender	**1·38**	**1·16, 1·65**	**1·37**	**1·15, 1·63**	0·07
FSM entitlement	**0·41**	**0·33, 0·50**	**0·40**	**0·33, 0·49**	
School-level FSM	**0·99**	**0·98, 1·00**	**0·99**	**0·98, 1·00**	
Sweets and crisps	**1·38**	**1·28, 1·49**	**1·41**	**1·30, 1·53**	
Individual-level demographics & school-level FSM & fruit and vegetables					
Gender	**1·44**	**1·20, 1·71**	**1·39**	**1·16, 1·65**	0·07
FSM entitlement	**0·39**	**0·32, 0·48**	**0·39**	**0·32, 0·47**	
School-level FSM	**0·99**	**0·98, 1·00**	**0·99**	**0·98, 1·00**	
Fruits and vegetables	**1·32**	**1·20, 1·46**	**1·22**	**1·12, 1·32**	

ICC, intra-cluster correlation coefficient. Significant results are highlighted in bold.

* Reference category for gender=male.

† FSM entitlement refers to those students from low-income families who are, therefore, eligible to receive free school meals at lunchtime (for the individual-level FSM variable, no=0 and yes=1).

‡ School-level FSM refers to the percentage of students within the whole school who are from low-income families and are, therefore, eligible to receive free school meals at lunchtime.

### Proximity of the dietary measures and examination date

Table 3 shows that among the nested cohort of children who provided measures at baseline and follow-up, the association of breakfast consumption with educational performance was stronger where the measure of breakfast consumption was more proximal to the collection of educational outcomes data (OR=2·02 for follow-up compared with OR=1·61 for baseline). For number of healthy breakfast items consumed, number of unhealthy breakfast items consumed, number of sweets and crisps consumed and number of fruit and vegetable portions, associations were similar whether using the proximal or more distal measure. As with the whole group, all dietary measures were associated with educational performance except for the consumption of unhealthy breakfast items.

**Table 3:** Nested cohort analysis (*n* 1216) to investigate the associations between dietary behaviours and improved educational outcomes among Year 5 and 6 students aged 9–11 years participating in the cluster-randomised controlled trial of the Welsh Government’s Primary School Free Breakfast Initiative for whom dietary behaviour is available at baseline and follow-up

	OR	Lower CI	Upper CI
Consumption of breakfast			
Baseline	**1·61**	**1·24**	**2·47**
Follow-up	**2·02**	**1·44**	**2·84**
Healthy breakfast items			
Baseline	**1·11**	**1·03**	**1·19**
Follow-up	**1·11**	**1·03**	**1·19**
Unhealthy breakfast items			
Baseline	1·18	0·94	1·48
Follow-up	1·01	0·81	1·26
Sweets and crisps (rest of day)			
Baseline	**1·41**	**1·23**	**1·62**
Follow-up	**1·42**	**1·24**	**1·64**
Fruit and vegetables (rest of day)			
Baseline	**1·28**	**1·08**	**1·52**
Follow-up	**1·25**	**1·07**	**1·47**

Significant results are highlighted in bold.

### Intention-to-treat analysis of intervention effects on educational outcomes

Following the intervention, 71·2 % (*n* 632) of children in the intervention group and 73·7 % (*n* 691) in the control group achieved a ‘high’ level of educational outcome. Results from the multilevel analysis indicated that between-group differences were not significant (OR=0·87; 95 % CI 0·65, 1·16; *n* 1826). Hence, there was no evidence of an intervention effect on educational outcomes.

## Discussion

In the present study, breakfast consumption, number of healthy breakfast items consumed, number of sweets and crisps consumed and number of fruit and vegetable portions consumed were all associated with significantly better educational performance at baseline and follow-up. The association of breakfast consumption with SATs scores was stronger at follow-up (i.e. 6 months prior to SATs) compared with baseline (i.e. 18 months prior to SATs), although a significant association remained in both analyses. No evidence of an intervention effect on educational outcomes was observed.

While a substantial body of existing literature focuses on links between breakfast eating behaviours and acute measures of concentration and memory^(^ ^12^ ^)^, the present analysis shows a meaningful link between dietary behaviours and concrete measures of academic attainment, which could have practical implications. The finding that associations between breakfast consumption behaviours and academic performance within the nested cohort analysis remained regardless of whether measured 6 or 18 months prior to examination perhaps indicates that breakfast consumption behaviours are relatively stable at this age, prior to moving to secondary school. Moreover, the finding that this association between dietary factors and SATs scores for the whole sample was significant at both baseline and follow-up provides further evidence that breakfast consumption behaviours may be relatively stable at this age and suggests that there may be a longitudinal effect of breakfast consumption on educational performance.

The association observed between healthy breakfast items and SATs scores *v*. the lack of association between unhealthy items and SATs scores at baseline and follow-up is consistent with an emerging body of research which suggests that a breakfast consisting of foods with a lower glycaemic index, which release energy steadily throughout the morning, may have a positive effect on students’ cognitive functioning, health, school attendance and academic outcomes^(^ ^31^ ^–^ ^33^ ^)^. For example, Mahoney *et al*.^(^ ^33^ ^)^ conducted a highly controlled experiment that manipulated breakfast content among 9–11-year-old children once per week for three weeks, finding that both boys and girls who had consumed breakfast foods with a higher glycaemic index had improved spatial memory and girls to have improved short-term memory. A further study by O’Dea and Mugridge^(^ ^34^ ^)^ observed an association between habitual breakfast quality and literacy scores, but not with numeracy scores.

However, there was also a significant association between consumption of sweets and crisps (eaten later in the day) and SATs scores in our analysis. One possible explanation for this could be an effect on more undernourished children. If children who are undernourished increase their energy intake, regardless of what they are eating, hunger is likely to be decreased and concentration and subsequent academic achievement improved. This is demonstrated by a 6-month school breakfast programme that observed significantly improved mathematics grades in undernourished children whose nutritional status improved to ‘adequate’^(^ ^32^ ^)^. Notably, while only one in five children ate sweets or crisps at breakfast, demonstrating no association with academic performance, a vast majority of children ate these items later on in the day. Further research is required to investigate any difference in the effects of breakfast consumption on children from families of differing levels of deprivation and the mechanisms by which this may occur.

Earlier results from the trial, reported in this journal, indicate that the initiative played a significant role in reducing SES inequality in pupils’ consumption of breakfast, which has been shown to be a socially patterned behaviour^(^ ^14^ ^)^. Hence, universal as opposed to targeted free breakfast programmes could play an important role in reducing health and educational inequalities. For example, more affluent children are likely to simply exchange a healthy breakfast at home for a healthy breakfast at school, while poorer children may exchange a poorer-quality home breakfast for a healthy school breakfast, gaining more from this exchange^(^ ^14^ ^,^ ^25^ ^)^. However, the current analysis provides no evidence that breakfast consumption mediated the relationship between SES and educational outcomes. Hence, while the study provides some support for the notion that promoting breakfast might improve overall academic performance, it provides no evidence that improving breakfast consumption would reduce inequality in educational outcomes.

### Strengths and limitations

Major strengths of the present study include the large sample and the longitudinal design. The measure of diet used in the current study involved dietary recall of the whole previous day and breakfast on the day of reporting. There have been several problems identified with the measurement of breakfast due to ambiguity over how it is defined in terms of frequency, time of day and type of food^(^ ^12^ ^)^. However, the measure used in the present study has shown adequate validity and reliability by comparison to more expensive and labour-intensive 24 h recalls. A further strength of the study is the use of SATs scores as a measure of educational attainment. SATs scores provide a measure that may be comparable across developed countries where age-specific tests are consistently sat by schoolchildren. This is particularly important due to the fact that studies investigating the effect of breakfast have often relied on self-report^(^ ^35^ ^)^ or used varied and simplistic measures of academic achievement, such as tests of acute cognitive function^(^ ^24^ ^)^. Intention-to-treat analysis was hampered by the fact that some control schools may have set up the free school breakfast scheme before SATs tests were taken. However, this would have diluted any potential intervention effect, rather than eliminating it altogether.

## Conclusions

Overall, these results demonstrate the association between consumption of healthy breakfast items and academic outcomes among primary-school children, with a stronger association observed when breakfast consumption is more proximal to the SATs tests. They also demonstrate that the significant independent associations of school- and individual-level SES with educational outcomes are not altered by addition of terms of dietary behaviours. Hence, while the study provides some support for the notion that promoting breakfast might improve overall academic performance, it provides no evidence that improving breakfast consumption would reduce inequality in educational outcomes.

Future research should aim to understand the causal mechanisms by which breakfast consumption and other health behaviours may improve academic outcomes. While this survey was conducted with children of primary-school age, research has shown that breakfast consumption declines once children begin secondary school^(^ ^13^ ^)^. This suggests that an interesting avenue for further research would be to investigate the effects of any drop in breakfast eating behaviour in secondary school on trajectories in young people’s educational performance. In addition, measures of educational performance should be integrated into trials of interventions to promote breakfast consumption and other health behaviours, in order to understand how interventions might promote both educational and health benefits simultaneously^(^ ^8^ ^)^. Such results could be vital in helping to align health improvement with the ‘core business’ of schools^(^ ^5^ ^,^ ^6^ ^)^ and to argue for the implementation of universal as opposed to targeted interventions.
